# SNP rs7549881, near SGIP1 at 1p31.3, is significantly associated with digestive disorders and Parkinsonism in women

**DOI:** 10.1016/j.prdoa.2025.100391

**Published:** 2025-09-02

**Authors:** Steven Lehrer, Peter Rheinstein

**Affiliations:** a Department of Radiation Oncology, Icahn School of Medicine at Mount Sinai, New York, NY, United States; b Severn Health Solutions, Severna Park, MD, United States

**Keywords:** Cigarettes, Smoking, Neurodegeneration, Genetics, Digestive disorders

## Abstract

**Background:**

SGIP1 (SH3GL Interacting Endocytic Adaptor 1) at 1p31.3 has been implicated in early-onset Parkinsonism in a rare familial case. We investigated the nearby intronic SNP rs7549881 (A > G, MAF 0.43) for association with Parkinson’s disease (PD) and other traits in the UK Biobank.

**Methods:**

A total of 385,629 UK Biobank participants with ≥7 years of education were included. Logistic regression adjusted for age, sex, smoking, and constipation evaluated the association between rs7549881 and PD. PheWAS was performed using PheWeb to identify additional phenotypic associations.

**Results:**

Among females, 26.0 % of rs7549881 GG homozygotes had PD, compared to 22.2 % without PD (p = 0.004). No significant genotype-PD association was observed in males. Logistic regression showed that age (OR 1.14), male sex (OR 1.83), and constipation (OR 4.73) increased PD risk, while smoking was protective (OR 0.75). GG genotype conferred increased PD risk versus AA (OR 1.21, p = 0.015). PheWAS identified significant associations with gastrointestinal phenotypes, including functional digestive disorders and noninfectious gastroenteritis.

**Conclusion:**

SNP rs7549881 is associated with PD in females and with gastrointestinal disorders across both sexes. These findings reinforce the role of SGIP1 in synaptic endocytosis and suggest its contribution to gut-brain axis dysfunction in PD.

## Graphical abstract

**Figure:**
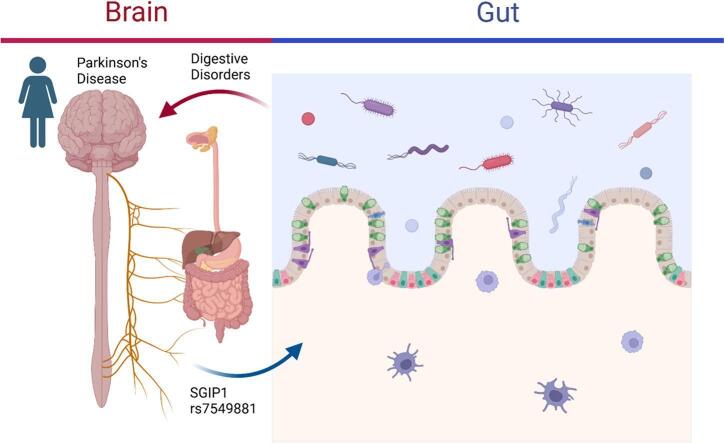

A recent study identified a variant in the SH3GL Interacting Endocytic Adaptor 1 (SGIP1, 1p31.3) gene linked to early-onset Parkinsonism. This variant was discovered in 2 sisters in an Arab family with a history of Parkinson’s symptoms that appeared at a young age. The SGIP1 gene had not been previously associated with Parkinsonism, a group of disorders characterized by motor dysfunction and cognitive decline, including Parkinson’s disease (PD). To study the effects of this variant, researchers created a fruit fly model lacking SGIP1. These flies exhibited movement difficulties and degeneration in brain cells, mirroring Parkinsonism symptoms. The variant caused defects in synapses. Specifically, the variant impaired protein recycling at the synapse, disrupting synaptic proteostasis, which is crucial for maintaining healthy neurons [1].

The SGIP1 gene is involved in regulating endocytosis, a process critical for cellular function and synaptic transmission in neurons. It plays a role in clathrin-mediated endocytosis, which allows cells to internalize molecules like neurotransmitters and membrane receptors. SGIP1 interacts with proteins involved in membrane dynamics and synaptic vesicle recycling, which are crucial for maintaining synaptic communication between neurons. Variants in SGIP1 can disrupt these processes, leading to synaptic dysfunction, which has been linked to neurodegenerative diseases such as early-onset Parkinsonism. The proper function of SGIP1 is therefore essential for healthy neuronal signaling and synaptic plasticity [2].

In the current study, we used data from UK Biobank to assess the relationship between SGIP1 at 1p31.3 and PD by studying a nearby common polymorphism, rs7549881 at 1p31.3. This variant was selected because it is a common intronic polymorphism near SGIP1, previously untested in Parkinson’s GWAS, making it of interest given the prior report of Decet et al of a monogenic SGIP1 loss-of-function variant. Decet et al. described a rare monogenic recessive loss-of-function variant, while the present study investigates common genetic susceptibility [1]. We then performed a phenome wide association study (PheWAS) of rs7549881 to further assess rs7549881.

## 1 Methods

The UK Biobank is a large-scale biomedical database and research resource containing in-depth genetic, lifestyle, and health information from around 500,000 participants. Launched in 2006, it is one of the most significant health studies in the world. The project collects data on various health measures such as blood pressure, physical activity, cognitive function, and genetic sequences, as well as information from medical records and biological samples (blood, urine, saliva).

The primary goal of the UK Biobank is to improve the prevention, diagnosis, and treatment of a wide range of serious and life-threatening illnesses, including cancer, heart disease, stroke, and dementia. Its data is available to approved researchers worldwide, making it a valuable resource for understanding the genetic and environmental factors that contribute to disease. The study's vast scale allows researchers to explore patterns and correlations at an unprecedented level, leading to numerous discoveries in public health and medicine.

UK Biobank has approval from the Northwest Multi-center Research Ethics Committee (MREC) to obtain and disseminate data and samples from the participants, and these ethical regulations cover the work in this study. Written informed consent was obtained from all participants. Details can be found at www.ukbiobank.ac.uk/ethics.

Our UK Biobank study was approved as UKB 57245 (SL and PHR). Our analysis included all subjects with PD that occurred either before or after participant enrollment and was recorded in the UK Biobank database using self-reported data and the International Classification of Diseases (ICD10, ICD9). We restricted our analysis to subjects with at least 7 years of education, since subjects with less education could have problems with the touch screen self-entry data system.

In the UK Biobank, constipation is defined based on responses to specific health-related questionnaires. The definition often relies on self-reported data from participants, which may include self-reported diagnosis, where participants might indicate if they have been diagnosed with constipation by a healthcare provider; bowel habit questions with specific survey questions regarding bowel habits, such as the frequency and difficulty of bowel movements, for example frequency of bowel movements (e.g., less than three times per week) and symptoms such as straining, incomplete evacuation, or hard stools; and linked clinical data, identifying constipation through linked electronic health records, using diagnostic codes such as ICD-10 K59.0 (constipation).

We studied the relation to PD of SNP rs7549881,1p31.3, a common single nucleotide intron variant, A > G, minor allele frequency 0.43, 201 base pairs. Data processing was performed on Minerva, a Linux mainframe with Centos 7.6, at the Icahn School of Medicine at Mount Sinai. We used PLINK, a whole-genome association analysis toolset, to analyze the UKB chromosome files. Statistical analysis was done with SPSS 26.

We then performed PheWAS (Phenome-Wide Association Study), a method used to explore the associations between genetic variants (typically single nucleotide polymorphisms or SNPs) and a broad range of phenotypes or disease traits. Unlike the more familiar GWAS (Genome-Wide Association Study), which focuses on finding genetic variants linked to a specific trait or disease, PheWAS does the opposite: it starts with a genetic variant, in this case rs7549881, and then looks across multiple phenotypes to see what associations exist.

PheWAS helps identify new connections between genetic variants and various diseases or traits, potentially uncovering pleiotropy, where one genetic variant affects multiple outcomes. By leveraging large datasets, such as electronic health records (EHRs) or biobanks like the UK Biobank, PheWAS can analyze many phenotypes simultaneously, leading to insights into the genetic basis of complex diseases, drug repurposing opportunities, and personalized medicine. This approach has been instrumental in discovering unexpected links between genetic variants and conditions, further deepening our understanding of the genetic architecture of human health​.

To perform PheWAS of rs7549881 we used the Phenome-Wide Association Study Browser PheWeb, a tool that allows researchers to explore the results of PheWAS analyses. PheWeb provides an interactive platform to visualize the associations between genetic variants (typically SNPs) and a wide array of phenotypes derived from large-scale biobank data, such as the UK Biobank and other genomic data resources.

## 2 Results

Data from 385,629 subjects was analyzed. PD patients were aged 63 ± 5 (mean ± SD). 95 % of subjects were white British.

Table 1 shows presence of Parkinson’s Disease versus rs7549881 genotype in female and male subjects. 26 % of female subjects homozygous for the minor allele (GG) of rs7549881 had PD. 22.2 % of female subjects homozygous for the minor allele (GG) of rs7549881 did not have PD. This difference was significant (p = 0.004, two tail Fisher exact test). The effect of rs7549881 genotype on male subjects was not significant (p = 0.183).

**Table 1:** Presence of Parkinson’s Disease versus rs7549881 genotype in female and male subjects. 26 % of female subjects homozygous for the minor allele (GG) of rs7549881 had PD. 22.2 % of female subjects homozygous for the minor allele (GG) of rs7549881 did not have PD. This difference was significant (p = 0.004, two tail Fisher exact test). The effect of rs7549881 genotype on male subjects was not significant (p = 0.183).

	Genotype				Total	p value
Female	GG	Count	46,387	128	46,515	0.004
		% Within parkinson	22.20 %	26.00 %	22.20 %	
	AG	Count	102,995	256	103,251	
		% within parkinson	49.30 %	51.90 %	49.30 %	
	AA	Count	59,667	109	59,776	
		% Within parkinson	28.50 %	22.10 %	28.50 %	
Total		Count	209,049	493	209,542	
		% Within parkinson	100.00 %	100.00 %	100.00 %	
Male	GG	Count	38,504	198	38,702	0.183
		% Within parkinson	22.00 %	24.60 %	22.00 %	
	AG	Count	86,356	377	86,733	
		% Within parkinson	49.30 %	46.80 %	49.30 %	
	AA	Count	50,420	231	50,651	
		% Within parkinson	28.80 %	28.70 %	28.80 %	
Total		Count	175,280	806	176,086	
		% Within parkinson	100.00 %	100.00 %	100.00 %	

Table 2 shows multivariate analysis of rs7549881/PD data with logistic regression. Male sex was associated with increased risk of PD (odds ratio O.R. 1.828, p < 0.001). Each year of age was associated with increased risk of PD (O.R. 1.141, p < 0.001). Cigarette smoking was associated with decreased risk of PD (O.R. 0.748, p < 0.001). Constipation was associated with increased risk of PD (O.R. 4.726, p = 0.001). rs7549881 GG genotype compared to AA genotype was associated with increased risk of PD (O.R. 1.208, p = 0.015).

**Table 2:** Multivariate analysis of PD data with logistic regression. Male sex was associated with increased risk of PD (odds ratio O.R. 1.828, p < 0.001). Each year of age was associated with increased risk of PD (O.R. 1.141, p < 0.001). Cigarette smoking was associated with decreased risk of PD (O.R. 0.748, p < 0.001). Constipation was associated with increased risk of PD (O.R. 4.726, p = 0.001). rs7549881 GG genotype compared to AA genotype was associated with increased risk of PD (O.R. 1.208, p = 0.015). (95 % L.B. 95 % confidence interval lower bound, U.B. 95 % confidence interval upper bound).

	95 % L.B.	O.R.	95 % U.B.	p value
Sex	1.634	1.828	2.044	<0.001
Age	1.130	1.141	1.152	<0.001
Smozking	0.647	0.748	0.865	<0.001
Constipation	1.933	4.726	11.551	0.001
AA vs GG	1.037	1.208	1.408	0.015
GA vs GG	0.927	1.057	1.205	0.417

PheWas of rs7549881 showed that the most significant associations were functional digestive disorders, personal history of diseases of the digestive system, and non-infectious gastroenteritis.

PheWas showed that the top 4 significant associations are digestive: functional digestive disorders, personal history of diseases of digestive system, noninfectious gastroenteritis, nausea and vomiting.

## 3 Discussion

The **SGIP1 gene** encodes a protein that plays a crucial role in endocytosis, particularly in neurons of the brain. One of its primary functions is participation in clathrin-mediated endocytosis, a process in which cells internalize molecules such as nutrients and neurotransmitters by engulfing them in clathrin-coated vesicles [3]. SGIP1 interacts with proteins including Eps15 and endophilins, which are essential components of the endocytic machinery, and through these interactions it facilitates the formation of clathrin-coated pits and vesicles [4]. SGIP1 is also highly expressed in neurons, where it is critical for the efficient recycling of synaptic vesicles—the small sacs that store neurotransmitters released during synaptic transmission. By enabling the rapid retrieval and recycling of these vesicle components, SGIP1 ensures sustained and efficient communication between neurons [1]. In addition, SGIP1 influences membrane curvature, an important feature of vesicle formation and trafficking, through its ability to bind phosphoinositides, a class of signaling lipids that regulate membrane dynamics [4].

Given these functions, SGIP1 has potential implications in disease. Variants in the gene have been linked to early-onset parkinsonism, where disruption of synaptic vesicle recycling may lead to impaired neurotransmission and neuronal degeneration [1]. Beyond neurodegeneration, abnormalities in endocytic processes involving SGIP1 can also affect neuronal development and connectivity, suggesting a possible role in neurodevelopmental disorders [5].

The SGIP1 gene is essential for maintaining efficient neuronal communication through its role in endocytosis and synaptic vesicle recycling. By facilitating the internalization and recycling of synaptic components, SGIP1 supports the rapid and repeated firing of neurons necessary for normal brain function. Ongoing research continues to investigate its broader implications in neurological diseases and how targeting SGIP1 pathways might offer new therapeutic strategies.

Although SGIP1 has not been notably associated with the gastrointestinal (GI) tract, the connection of nearby SNP rs7549881 with functional digestive disorders is not surprising. Indeed, a recent genome-wide pleiotropic analysis demonstrated a role of the gut-brain axis in the shared genetic etiology between gastrointestinal tract diseases and psychiatric disorders [6]. This association is important because of the close relationship between digestive disorders and PD.

GI disorders are a common non-motor symptom of PD and are linked to the disease's development and progression. Up to 80 % of PD patients experience constipation, which usually begins before other symptoms. Additional common GI symptoms include bloating, nausea, vomiting, and gastroparesis. Some research suggests that the gut may be the initial site of pathological changes in PD. The accumulation of Lewy pathology in the enteric nervous system can lead to impaired motility and constipation [7]. Moreover, people with PD have reduced levels of some beneficial bacteria, like Prevotella, Faecalibacterium, and Roseburia, while others, like Bifidobacterium and Lactobacillus, are increased [8].

The study has several strengths. It leverages the UK Biobank, one of the largest deeply phenotyped and genotyped cohorts worldwide, enabling robust analysis of both primary associations with Parkinson’s disease and pleiotropic effects through PheWAS. The large sample size provides statistical power to detect modest genetic effects.

However, limitations should be acknowledged. Parkinson’s disease status was determined using both self-report and ICD-coded diagnoses, which may introduce misclassification. The cohort is predominantly of white British ancestry, limiting generalizability to more diverse populations. Furthermore, the study lacks an external replication dataset, which would be necessary to confirm the observed associations. Finally, while the PheWAS highlights links to gastrointestinal traits, causality cannot be inferred from these cross-sectional associations.

In conclusion, the SGIP1 gene at 1p31.3 plays a critical role in endocytosis and synaptic vesicle recycling, essential processes for maintaining healthy neuronal communication. The SGIP1variant previously identified in 2 young sisters has provided new insights into its involvement in early-onset Parkinsonism, highlighting how synaptic dysfunction contributes to neurodegeneration [1]. Our study above demonstrates an association between nearby SNP rs7549881 at 1p31.3 and Parkinson's disease, particularly in female subjects, as well as links to functional digestive disorders. These findings emphasize the potential for SGIP1 and rs7549881 to serve as therapeutic targets, not only in Parkinsonism but also in addressing the broader gut-brain axis, which may influence both neurological and gastrointestinal health. Ongoing research could lead to novel therapeutic approaches for neurodegenerative diseases.

Data sources

Data available from UK Biobank after approved application

## CRediT authorship contribution statement

**Steven Lehrer:** Formal analysis, Data curation, Conceptualization. **Peter Rheinstein:** Formal analysis.

## Funding

None.

## Declaration of competing interest

The authors declare that they have no known competing financial interests or personal relationships that could have appeared to influence the work reported in this paper.
